# Humans may not have a uniquely enhanced sequence memory: sequence discrimination is facilitated by causal–logical framing in humans and chimpanzees

**DOI:** 10.1098/rsos.250236

**Published:** 2025-07-16

**Authors:** Eva Reindl, Amanda M. Seed, Robert A. Barton, Topaz Francis-Costa, Rachel L. Kendal

**Affiliations:** ^1^ Department of Anthropology, Durham University, Durham, UK; ^2^ School of Psychology and Neuroscience, University of St Andrews, St Andrews, UK; ^3^ School of Social Sciences, University of Mannheim, Mannheim, Germany

**Keywords:** sequence cognition, sequence discrimination learning, sequence memory, comparative cognition

## Abstract

Humans have been suggested to possess uniquely enhanced memory for sequences, based on sequence discrimination learning (SDL) tasks involving arbitrarily ordered sequences with no functional connection to outcomes. Such tasks underestimate animals’ SDL as they lack affordances of real-world situations. We tested whether stimuli causally connected to outcomes facilitate SDL. A total of 13 chimpanzees, 24 capuchin monkeys, 23 squirrel monkeys, 77 adult and 239 juvenile humans completed an AB versus BA, BB and AA task. Humans discriminated causal sequences better than arbitrary ones and one chimpanzee succeeded in the causal frame condition after 324 trials, suggesting that performance gaps in previous studies are partly due to the arbitrariness of sequences (monkeys mostly failed the training). Without causal framing, children found the task difficult until 10–11 years of age. The sequence memory hypothesis needs to be evaluated with a broader set of tasks and account for cultural scaffolding of participants’ understanding of task requirements.

## 1. Introduction

Processing sequences is a ubiquitous demand in human lives, be it in language, complex action sequences such as tool use, dance, music or cooking, social interactions, spatial navigation, episodic memory or mathematics [1,2]. It has recently been suggested that the capacity to process, remember, produce and predict complex sequences is uniquely enhanced in humans [2–8]. The cognitive abilities underlying this capacity and the extent to which it may be present in other species are still debated [3–6]. Some hypotheses point to changes in memory [2,5,9–11]. For example, the *memory for stimulus sequences* hypothesis proposes a qualitative difference between humans and other animals specifically in the way sequences are encoded and remembered [5]. It suggests that, while humans form precise representations of the order of items within a sequence and can tell different sequences apart quickly, non-human animals (henceforth animals) do not remember the relative position of items faithfully and confuse even short sequences easily, even after hundreds of exposures (electronic supplementary material, tables S3 and S4). It is not claimed that animals are completely ‘blind to sequences’ or that it is impossible for them to tell an AB from a BA sequence apart (see [12]), but that animals do not represent sequences *faithfully*, instead relying on strategies such as the strength of memory traces for individual components. The main line of evidence for this theory comes from sequence discrimination learning (SDL) tasks [2].

## 2. The sequence discrimination learning paradigm

In these tasks, participants are presented with two or more different sequences (one at a time) and trained to discriminate between them. Sequences can be auditory (e.g. tunes or bursts of white noises), visual (e.g. coloured squares on a screen or light flashes in a testing chamber) or olfactory. Participants learn to respond to a specific sequence (e.g. comprising elements A, then B) in one way and to show a different response to sequences comprising the same elements in different orders (e.g. BA or AA). Note this paradigm is concerned with sequence *perception*. It does not require participants to produce sequences themselves (as happens in simultaneous chaining [13] or observational studies [14]) as such does not necessarily require memory or representation of the sequence.

Most SDL studies have used short sequences of two to three stimuli (e.g. AB versus BA; AB versus BA, BB and AA; ABC versus AAB) and involved songbirds and pigeons, with fewer studies on rats, monkeys, bonobos, dolphins or dogs (electronic supplementary material, table S1; [2,7]). Only a handful of studies with humans exist as these discriminations are trivially easy for adults (electronic supplementary material, table S2). In contrast, animals take hundreds, often thousands, of trials to discriminate between such sequences. For example, capuchin monkeys required 820–2520 trials to reach 90% success [15] (electronic supplementary material, table S3), and bonobos did not discriminate in an AB versus BA, BB and AA task within 2400 trials [7]. From these data, proponents of the memory for stimulus sequences hypothesis concluded, while emphasizing the importance of culture and social learning for the development of human sequencing skills, that humans have a genetic basis for ‘enhanced abilities to represent sequential information’ [5, p. 19].

However, the existing body of SDL studies has three main limitations which render premature the conclusions that (i) humans differ in their SDL performance from all other animals and (ii) such differences reflect qualitative (and genetically based) differences specifically in sequential information processing. Below, we briefly outline these issues: the generalization problem, inference problem and data skewness problem.

### 2.1. The generalization problem

Existing SDL studies have mainly focused on using ‘arbitrary sequences’ [2,7] (definition below), with two consequences: first, arbitrary sequences might underestimate animals’ SDL performance as they might be especially difficult to process; second, it is unclear whether animals’ reported difficulties in SDL tasks apply to all types of sequences or only to those arbitrary sequences. The term ‘arbitrary’ has been used to refer to two different, but related, characteristics of sequences: (i) the extent of order or relation of the stimuli within a sequence, i.e. to what extent the stimuli themselves dictate their position in a sequence [16,17] and (ii) the way sequences are linked to required participant responses or task outcomes. Regarding the order of stimuli, sequences have been categorized as *enabling* (i.e. when one step can logically only happen after another step), *monotonic* (when stimuli are ordered along a dimension, e.g. circles increasing in size), or—as most often used in SDL tasks (electronic supplementary material, table S3)*—arbitrarily ordered* (when there is nothing inherent in the sequence that carries information about the ‘correct’ order of its stimuli). Enabling sequences may be better remembered because their stimuli can more easily be perceived as a unit and/or because they often lead to more interesting outcomes [18]. Indeed, data indicate processing arbitrarily ordered sequences is more difficult than monotonic ones for orangutans [17] and more difficult than enabling ones for children [17,18]. Regarding sequence linkage to outcomes or responses, in SDL studies, the order of stimuli within sequences is typically not informative about which response is required from the participant. For example, bonobos [7] needed to press a button on the left after seeing a screen turn blue then yellow (AB) but press a button on the right after seeing the screen turn yellow then blue (BA) or after the remaining sequence variations (BB and AA). Whether the left button press was required after an AB or BA sequence was ‘arbitrarily’ determined by the experimenter and not inherently linked to the stimulus order. Animals’ difficulties in such tasks may be impacted by this *non-functional connection* between the order of stimuli and the required response because ‘stripping meaning away from stimulus items […] can exclude features that are crucial to learning’ [19, p. 3]. Indeed, we know that while non-human primates can learn to make decisions based on cues that are arbitrarily related to a reward, they learn substantially faster if ‘discriminative cues ha[ve] functional relevance to the outcome’ ([20, p. 186]; see also [21–26]). In sum, it is unclear to what extent animals’ difficulties in SDL tasks also apply to sequences that are more meaningfully ordered (e.g. by a causal or social relation) and have a more functional connection to a desired response.

### 2.2. The inference problem

Animals’ difficulties in SDL tasks may stem from differences in inductive biases, making the SDL starting point for humans and other animals less comparable. Existing SDL tasks conflate measuring abilities to *process and remember sequences* with abilities and proclivities to infer that one should *attend to the stimuli as a sequence rather than as individual units*. Differentiating between these sources of difficulty is important if one argues that animals struggle with only *one* aspect (e.g. the ability to remember sequences [7]). Sequences of perceptual stimuli are probably less prevalent in most animals’ environments than in humans’ [2], so it is possible animals do not have a spontaneous tendency to consider stimuli as sequences even though they might be able to do so. Human participants typically are exposed to sequencing tasks outside of experimental contexts and therefore readily grasp what problem to solve, whereas for non-human participants, there is no cue to initially orient them to the nature of the task. Thus, animals’ difficulties in SDL tasks could be partly due to a weaker inductive bias to process stimuli as a sequence, whereas humans arrive at the task well primed due to their life experiences and the cultural ubiquity of information presented as sequences. In short, experiments with arbitrary sequences may have low ecological validity.

A similar argument—that inductive biases can partly explain population differences in experimental tasks—has recently been made for the relational match to sample task (RMTS [27–32]). Preschool children and animals find this task difficult, with children preferring to match based on object identity than on relation [28,33]. However, priming 4-year-olds with a simpler match to sample (MTS) task, without explicit verbal instructions, enabled them to pass a subsequent RMTS task, suggesting training changed their initial bias towards using same/different as a basis for matching [28]. Similarly, embedding the RMTS task into a causal context (using a narrative) significantly improved 4-year-olds’ performance [34]. Moreover, while MTS tasks can be learned by many species, it often takes hundreds or thousands of trials to do so [35–38]. Differences in spontaneous attention toward object similarities rather than an inability to identify relational similarities *per se* have been discussed as contributing factors to performance differences between humans and other animals [31,39–41], further highlighting species differences in inference making. In essence, non-human participants may do poorly simply because they do not understand what ‘question’ they are being asked, unlike human participants who bring cultural resources to such tasks. Thus, study populations may substantially improve performance when the demand of inferring basic rules of the task is decoupled from the actual capacity in question.

### 2.3. The data skewness problem

Until recently, SDL studies with non-human great apes and human children were lacking. Existing studies compared animals’ performance solely with adult humans [2]. Finding large differences between human and other species might not be surprising, given we know that almost all aspects of human cognition are affected and often transformed by culture [42]. The proponents of the memory for stimulus sequences hypothesis themselves emphasize that many of humans’ sequencing abilities are culturally learned (e.g. counting, mathematics, music, reading and writing) [5]. Human sequencing abilities may not be much different to that of other primates early in development [42]. Studies with children can illuminate the extent to which differences are due to different developmental starting points or trajectories [43]. Second, more studies with non-human great apes are needed, as it is possible their sequence memory falls between that of humans and other animals: compared with monkeys, great apes have a larger cerebellum (relative to both body size and to the neocortex), and this brain region has been linked to sequence learning [44,45].

### 2.4. The current study

We suggest that differences in processing and remembering sequences between humans and animals may be smaller than previously claimed because existing SDL tasks have used only specific types of sequences (not generalizable) that might have been especially difficult to process for animals (underestimating performance) and thus posed an inference problem in addition to the sequence learning demand and because data points from non-human great apes and children have been largely missing. The current study aimed to provide new evidence on SDL using a task grounded in the causal logic of real objects (rather than an arbitrary string of artificial stimuli), providing causal connections between the stimuli within a sequence and between the stimuli and required response. We also include crucial unstudied groups in this paradigm: chimpanzees and human children.

We investigated performance of zoo-housed chimpanzees, capuchin and squirrel monkeys, as well as human adults and children (3–11 years, United Kingdom) in a two-stimuli sequence SDL task (AB versus BA, BB and AA) [2,7,46]. We used a two-choice rather than a Go/No-go paradigm, as we deemed it more intuitive and engaging for participants [7]. We aimed to investigate whether the presence of a causal frame would boost SDL in our non-human participants to such an extent that they could solve the discrimination in substantially fewer trials than previously reported [2]. We judged a theoretically interesting, substantial boost in SDL to be if animal participants reached the discrimination learning criterion (defined below) in fewer than 300 test trials (electronic supplementary material, S2.7.1.). Adults were included as a reference point and to test the hypothesis that the presence of a causal frame would facilitate SDL in humans also. We included children of a large age range to provide lacking developmental data on SDL [5], although note that children’s understanding of perceptual sequences has been investigated from other perspectives (e.g. event representations [47,48], memory and imitation of action sequences [49–51], sensitivity to adjacent and non-adjacent relations within sequences [52]).

**Figure 1.**
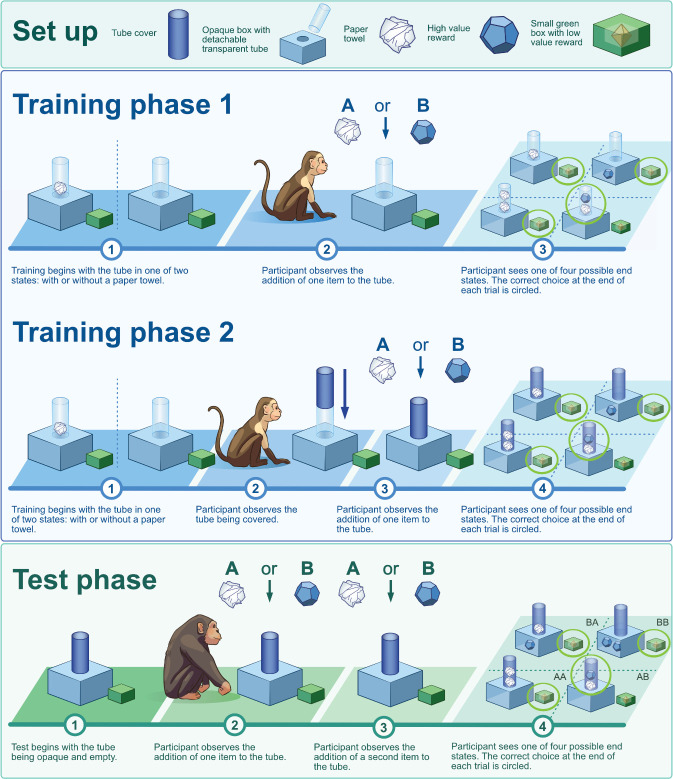
The set-up and procedure of the ‘reward and blockage sequence (RABS)’ task. Figure designed by Emily Green for Science Graphic Design. Notes. For all animal participants (squirrel monkeys, capuchin monkeys and chimpanzees), the study started with food preference tests (not depicted here, see OSF page). Before starting training with the RABS apparatus, all participant groups were administered a choice (opt-out) training to familiarize them with the basic structure of the task (not depicted here, see main text). Training phase 1 was administered to non-human animals (squirrel monkeys, capuchin monkeys and chimpanzees) only but skipped for human participants to save time. Training phase 1 had four goals: (i) to prime animals to attend to the combination of objects in the tube of the RABS apparatus; (ii) to familiarize them with the four possible endstates of the tube: empty tube, one paper towel in tube, two pieces of paper towel in tube, paper towel and food reward in tube; (iii) to help them learn which endstate required them to point to the apparatus to receive the high-value food reward (i.e. when there was a paper towel and food in the tube) and which required them to point to the opt-out box to receive the lower value food reward (the other three sequence types); (iv) for participants in the causal frame condition, to give them the opportunity to learn about the causal relations between the objects in the RABS tube. Training phase 1 required participants to attend to the type, number and order of objects within the tube but not to attend to the experimenter’s actions. Training phase 2 was administered to squirrel monkeys, capuchin monkeys and human participants but skipped for chimpanzees for time budget reasons. The procedure of training phase 2 was the same as in phase 1 with the only difference being that the experimenter added an opaque sleeve to the tube before the demonstration of a single action. The goal of training phase 2 was to familiarize participants with pointing to a completely opaque apparatus (as was done in the test phase). The test phase was administered to chimpanzees and humans but not administered to monkeys due to their poor performance in the training phase (electronic supplementary material, S3.2): In each test trial, participants watched the experimenter carry out a sequence of two actions on the opaque RABS tube.

We created the *reward and blockage sequence (RABS) task* in which participants watch an experimenter carry out sequences of two actions on an opaque tube placed on top of a box (figure 1). One action is to add a high-value reward (food for non-human animals and tokens for human participants) to the tube (that will fall straight through the tube and be trapped inside the box). The second action is to add a blockage to the tube (a balled-up paper towel) which can stop the reward from falling into the box if inserted before the reward. Only the order ‘AB’ (paper towel, then reward) results in the reward remaining in the tube and thus being available to the participant. After the other possible sequences, the tube contains either one towel (BA), two towels (AA) or is empty (BB). After the demonstration, participants can point to the tube (correct response for AB trials) or to a green ‘opt out’ box where they receive a lower value reward (correct response in BA, BB and AA trials). In the *causal frame* condition, when the participant points to the tube, the experimenter detaches the tube and turns it upside down. After an AB sequence, this will cause the reward to fall out to be provided to the participant. In the *no-causal frame* condition, the experimenter does not detach the tube. Instead, they open a separate box that only contains a reward in AB trials. In the no-causal frame condition, the connection between the action sequence and the availability of the high-value reward is arbitrary, but no less predictive. The RABS task was developed as a novel approach to investigating SDL across species and is not presented as a finalized or even standardized test but one that informs future task designs that balance ecological validity and cognitive comparability. As we report transparently below and in the electronic supplementary material, some aspects of the task did not work as desired; most significantly the fact that this task version posed high non-sequencing-related demands (namely on inhibitory control and working memory). Example videos for all study groups apart from the children can be found on the project’s OSF page.

An understanding of solidity and support—i.e. that the towel stops the reward from falling—is not *required* to solve this task. It is possible to discriminate between the sequences through trial-and-error or rule learning, without understanding why the AB sequence requires one response, while the other sequences require another. However, experience with support and solidity can accelerate learning, particularly when differentiating between AB and BA. Great apes—and to a seemingly lesser extent monkeys—appreciate the solidity of physical objects: they understand that solid objects cannot move through other solid objects, can produce noise and—critically for this study—will come to a halt at solid barriers [24,53–59]. Therefore, given sufficient experience with the functional properties of the objects in our task (acquired during the training phases; see figure 1), we predicted that participants would be better able to discriminate between sequences in the *causal frame* than the *no-causal frame* condition (electronic supplementary material, S2.3).

## 3. Results

### 3.1. Chimpanzees

Chimpanzees received one training phase (phase 1; initially set to a maximum of 180 trials, but chimpanzees who failed continued to be tested in this phase) and the test phase (max 300 trials). Seven chimpanzees passed the training: five out of five in the causal frame condition (of which three passed within 180 trials), two out of five in the no-causal frame condition (both passed within 180 trials), indicating understanding to flexibly point to the opt-out box or RABS apparatus depending on the type, number and order of objects (paper towel and food reward) within the tube, and these chimpanzees then proceeded to the test phase. Proportions of chimpanzees passing the training phase did not significantly differ between conditions (Fisher’s exact test, *p* = 0.167), though note that cell sizes were consistently below five individuals. Chimpanzees in the causal frame condition needed significantly fewer trials to reach the training criterion within 180 trials than chimpanzees in the no-causal frame condition (negative binomial generalized linear model, *β* = 0.48, s.e. = 0.20, *z* = 2.45, *p* = 0.014; though again due to low sample sizes of three and two chimpanzees, respectively, this finding needs to be interpreted cautiously). Further results from the training phase see figure 1 and electronic supplementary material, S2.5.

**Figure 2.**
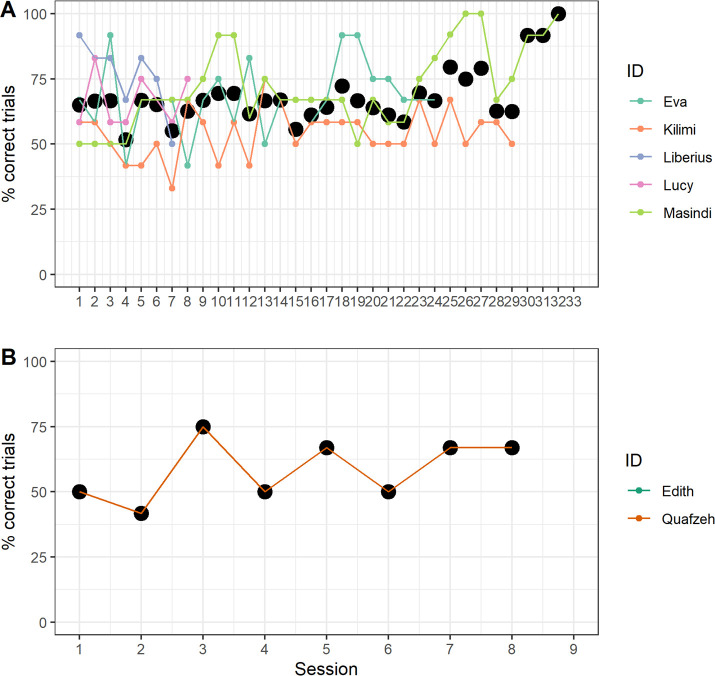
Percentage of correct trials per session in the test phase for chimpanzees in the causal frame (A) and no-causal frame (B) condition. The larger black dots indicate mean percentages across chimpanzees. Note that Edith only completed a few trials of session 1.

In the causal frame condition, three chimpanzees completed all 25 test sessions (300 trials), the others completed seven and eight sessions. No chimpanzee passed the test within 300 trials, though one (Masindi) passed in session 27 (after 324 trials; note that if a chimpanzee failed a training/test phase, we kept testing them in that phase until they passed or the study ended). In the no-causal frame condition, neither chimpanzee completed the test phase (one completed eight sessions and one a few trials of session 1; figure 2; table 1; electronic supplementary material, tables S7, S20 and S21). Therefore, no hypotheses could be tested, i.e. we could not make meaningful comparisons between chimpanzees’ performances between the two conditions (for details and exploratory analyses see electronic supplementary material, S3.2 and S3.3).

**Table 1.** Numerical summary of performance of all study groups in all administered study phases.

species	variable	phase						
		opt-out training	training 1		training 2		test	
			causal frame	no-causal frame	causal frame	no-causal frame	causal frame	no-causal frame
squirrel monkeys	no. of monkeys completing phase	22	7	NA	2	NA	NA	NA
	no. of monkeys reaching criterion	13 (59%)	2 (28%)*	NA	0 (0%)*	NA	NA	NA
	mean no. of trials needed to reach criterion (±s.d., min–max)	81 ± 47, 32−176	117 ± 64, 72−162	NA	/	NA	NA	NA
capuchin monkeys	no. of monkeys completing phase	20	10	NA	7^a^	NA	NA^f^(erroneously)	NA
	no. of monkeys reaching criterion	20 (100%)	10 (100%)	NA	1 (14%)*	NA	NA (erroneously)	NA
	mean no. of trials needed to reach criterion (±s.d., min–max)	38 ± 17, 32−96	108 ± 42.43, 72−198	NA	108	NA	NA (erroneously)	NA
chimpanzees	no. of chimpanzees completing phase	12^b^	5	5^d^	NA	NA	3^g^	0^f^
	no. of chimpanzees reaching criterion within 10 sessions (training)/25 sessions (test)	11 (92%)^c^	3 (60%)	2 (40%)^e^	NA	NA	0 (0%)	/
	*no. of chimpanzees reaching criterion including all administered sessions*	11 (92%)	5 (100%)**	2 (40%)*	NA	NA	1 (33%)**	/
	mean no. of trials needed to reach criterion (±s.d., min–max) within 10 sessions (training) / 25 sessions (test)	35 ± 6, 32−48	72 ± 18, 54−90	117 ± 38, 90−144	NA	NA	/	/
	*mean no. of trials needed to reach criterion (±*s.d.*, min–max) including all administered sessions*	35 ± 6, 32−48	166 ± 134, 54−360	117 ± 38, 90−144	NA	NA	324	/
human adults	no. of adults completing phase	77	NA	NA	39	38	39	38
	no. of adults reaching criterion	77 (100%)	NA	NA	no criterion	no criterion	3 (8%)	1 (3%)
	mean no. of trials needed to reach criterion (±s.d., min–max)	4.05 ± .45, 4−8	NA	NA	no criterion	no criterion	12 (fixed)	12 (fixed)
	mean percentage of correct trials	/	NA	NA	0.79 ± 0.10, 0.42-0.92	0.73 ± 0.14, 0.33−1	0.81 ± 0.15, 0.33-.1**	0.69 ± 0.18, 0.25−1*
human children	no. of children completing phase	239	NA	NA	114	125	114	125
	no. of children reaching criterion	no criterion	NA	NA	no criterion	no criterion	14 (12.3%)	2 (1.6%)
	mean no. of trials needed to reach criterion (±s.d., min–max)	/	NA	NA	no criterion	no criterion	12 (fixed)	12 (fixed)
	mean percentage of correct trials	/	NA	NA	0.73 ± 0.16, 0.33−1	0.65 ± 0.22, 0−1	0.78 ± 0.16, 0.42−1**	0.52 ± 0.15, 0.25−1*

Notes. One asterisk (*) highlights those phases in which performance was relatively low (monkeys: training phase; chimpanzees, humans: no-causal versus causal frame condition), two asterisks (**) highlight those phases in which performance was relatively good (chimpanzees, humans: causal-frame condition).

^a^ An additional four capuchin monkeys started but did not complete, Phase 2.

^b^ One additional chimpanzee (Rene) started but did not complete, the opt-out training.

^c^ One chimpanzee (Louis) did 30 sessions of opt-out training without passing criterion.

^d^ One additional chimpanzee (Sophie) started but did not complete the training.

^e^ One chimpanzee (Edith) was erroneously marked as having reached criterion after session 5 and moved to the test phase, while she should have continued to stay in the training phase; therefore, the mean number of trials needed to reach criterion in the no-causal frame condition is underestimated.

^f^ The experimenter failed to notice that one monkey (Cayenne) passed training phase 2 in session 6 and erroneously tested this monkey further up to session 10 instead of proceeding to the test phase.

^g^ Two additional chimpanzees started, but did not complete, the test phase (causal frame condition: Lucy, Liberius; no-causal frame condition: Quafzeh, Edith).

### 3.2. Adult humans

**Figure 3.**
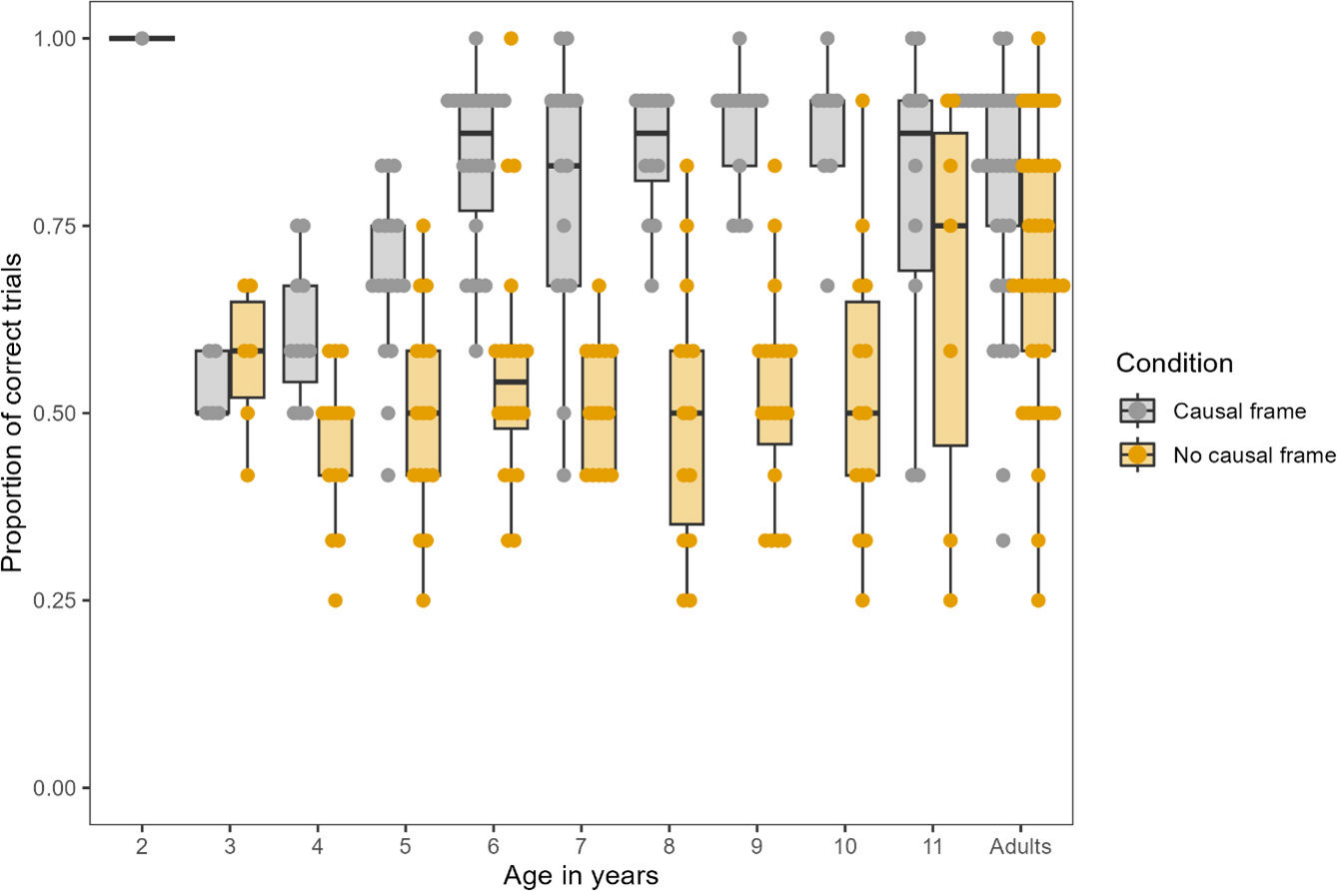
Average proportion of correct trials achieved in the test phase of the causal and no-causal frame condition by adults and children. The black midline in each boxplot represents the median, the upper and lower limits of the box the third and first quartile (75th and 25th percentile), respectively, the whiskers extend up to 1.5 times the interquartile range from the top of the box (or bottom, respectively) to the furthest data point within that distance.

Adults received one training phase (‘phase 2’, 12 trials) and one test phase (12 trials). The mean proportion of correct trials in the causal frame condition (0.81 ± 0.15, 0.33–1) was significantly higher than in the no-causal frame condition (0.69 ± 0.18, 0.25−1), *W* = 1035.5 and *p* = 0.002 (figure 3, electronic supplementary material, figure S35). There was no significant difference between conditions regarding the percentage of adults reaching the learning criterion (8% in the causal frame, 3% in the no-causal frame condition; two-proportions *z*-test, *χ*^2^(1) = 0.24, *p* = 0.313; electronic supplementary material, table S50).

### 3.3. Children

Children received one training phase (‘phase 2’, six trials) and one test phase (12 trials). Their mean proportion of correct trials in the causal frame condition (0.78 ± 0.16, 0.42−1) was significantly higher than in the no-causal frame condition (0.52 ± 0.15, 0.25−1), *W* = 12267 and *p* < 0.001. The percentage of children reaching the learning criterion was also significantly different between conditions (12.3% causal frame, 1.6% no-causal frame; two-proportions *z*-test, *χ*^2^(1) = 9.25 and *p* = 0.001).

Exploratory analyses investigated the effects of condition, age in years, sequence type (AB, BA, BB and AA) and the interaction between condition and age on trial-by-trial success with a generalized linear mixed model [60]. The interaction term significantly improved model fit (*χ*^2^(1) = 10.92, *p* = 0.001), so we proceeded by analysing the effect of age separately by condition.

In the causal frame condition, there was a significant, positive effect of age (*χ*^2^(1) = 22.58, *p* < 0.001) and a significant effect of sequence type (*χ*^2^(3) = 213.55, *p* < 0.001). Performance in the A (paper towel) B (token) and AA trials was not significantly different from each other (mean proportion of correct trials: AB: 0.87 ± 0.21; AA: 0.89 ± 0.26; *p* = 0.739; electronic supplementary material, table S53), and performance in both sequence types was significantly higher than in the BA (0.74 ± 0.35) and BB (0.42 ± 0.35) trials (all *p* < 0.001). Performance in BA trials was significantly higher than in BB trials (*p* < 0.001). That is, choosing correctly in the AB trials and in those in which no token was involved (AA) was comparably easy, BA trials were of intermediate difficulty and BB trials (two tokens being inserted) were the most difficult. We deemed part of the difficulty of BB trials to be because the first trial in the test phase was always a BB trial. We, therefore, reran the analyses with the first test trial removed (electronic supplementary material, S3.3.3.4.3)—the qualitative pattern of results did not change for the causal frame condition.

In the no-causal frame condition, there was also a significant effect of sequence type (*χ*^2^(3) = 53.98, *p* < 0.001) but no effect of age (*χ*^2^(1) = 1.73, *p* = 0.188), i.e. the task was comparably difficult for all age groups. As in the causal frame condition, performance in the AB (0.61 ± 0.25) and AA trials (0.53 ± 0.39) was of comparable difficulty (*p* = 0.193), though numerically lower than in the causal frame condition. Again, performance in the AB trials was significantly higher than in the BA (0.42 ± 0.37) and BB (0.37 ± 0.35) trials (both *p* < 0.001). While we initially found differences between the AA, BB and BA trials, the reanalysis with the first test trial removed showed that all opt-out trials (i.e. BB, BA and AA) were of comparable difficulty (see electronic supplementary material).

We also analysed the effect of condition separately by yearly age groups (electronic supplementary material, S3.3.3.4.2). For all, apart from 3- and 11-year-olds, performance in the causal frame condition was significantly higher than in the no-causal frame condition (figure 3). For 3-year-olds, both conditions were comparably difficult, i.e. the presence of a causal frame did not boost SDL (percentage correct in causal frame: 0.53 ± 0.04: no-causal frame: 0.57 ± 0.10). The average percentage of correct trials in the causal frame condition increased with age from 3 to 6 years and then remained at a high level. Performance in the no-causal frame condition stayed at the same level from 3 to 10 years; only at 11 years did children in the no-causal frame condition on average perform as well as those in the causal frame condition.

### 3.4. Monkeys

No monkeys entered the test phase because no squirrel monkeys and only one capuchin monkey (electronic supplementary material, S2.9.2) passed the second training phase. Thus, no hypotheses were tested. Performance in the training phases suggested that the set-up and procedure of the RABS task in its current version were not well suited to familiarize monkeys with the properties of the task and ensure they were able to discriminate between sequence types when only single actions were used (electronic supplementary material, S4.3).

## 4. Discussion

We showed that the arbitrary nature of sequences affects performance in SDL tasks: our adult and child participants were better able to discriminate between artificial stimulus sequences when there was a logical structure to at least one of the sequences (here a physical cause-and-effect relation between stimuli), which facilitated learning to respond differently to AB versus BA, BB and AA sequences. In addition, we obtained intriguing results for the chimpanzees, as we found one individual in the causal frame condition reached criterion after only 324 trials, thus performing substantially better than bonobos tested in an ‘arbitrary’ version of the AB versus BA, BB and AA task who did not reach criterion within 2400 trials [7], despite ample research experience and motivation (V Vinken 2023, personal communication). Thus, the difficulties animals have been reported to experience in past SDL studies may in part have been due to the absence of logical structures in the sequences. Accordingly, prior studies may have measured not only the *ability to process and remember* sequential information but also the *proclivity to infer* that the task was about processing stimuli as sequences (rather than as individual units; the ‘inference problem’). As has been argued in the context of *Artificial Grammar Learning* tasks, removing all meaningful (e.g. perceptual or semantic) relations between stimuli will probably remove features that are integral parts of sequence learning [19]. While investigating how species process arbitrarily ordered sequences that are also arbitrarily linked to outcomes has its own merits [2], we need to study richer, more ecologically valid types of sequences to gain a more complete understanding of species’ sequence learning and differences therein. This is especially important if evidence from SDL and similar tasks is used to claim that animals lack faithful memory for sequences *of all kinds* (the ‘generalization problem’) and that processing sequences is a key cognitive bottleneck differentiating humans from all other animals [2,61].

Animals probably do not understand in the same way as humans that they are being ‘asked’ to solve a sequence task, even when highly motivated and experienced with experimental tasks in general. This is especially the case when any supporting logical framing is missing, as this inference then requires an even bigger ‘jump’ in hypotheses space. For example, the bonobos in [7] were administered MTS tasks before being tested on an SDL task, and the experience with the former, in which they had to respond to single stimuli, could have been counterproductive for switching attention to the sequence level on the SDL task. Similarly, in the trace model test of the same study, sequence trials were interleaved with MTS trials, which again might have made it difficult for the bonobos to learn that the rules in the SDL task were different.

More generally, this inferential jump is larger for animals than for humans because of their different life experiences. Humans typically grow up in rich cultural contexts in which temporal sequences play a prominent role in many domains of life [2], and so cultural experiences equip them with a strong bias to process incoming information as sequences. Perceptual sequences are less prevalent in most animals’ environments and so it is possible that animals are less prone to consider stimuli as sequences despite a capacity to do so. Humans are also generally better in inferring the goals, expectations and implicit rules of laboratory tasks due to their rich experiences with social learning, teaching and testing [28,29,34]. Nevertheless, even adult humans, arguably the ‘super sequencers’ in the animal kingdom, who are equipped with a strong proclivity to infer patterns from sequential data [6,62], need to make this inferential jump in the absence of any framing, as evidenced by the difference between the causal and no-causal frame conditions. For children, we found that having to draw this inference limits their SDL performance, reaching adult-level performance only by 11 years of age.

One chimpanzee (Masindi) in the causal frame condition, in which we used cause-and-effect relations between stimuli, succeeded in discriminating between AB and BA, BB and AA in just over 300 trials, and thus learned substantially faster than recently tested bonobos in an arbitrary, non-physical, version of the AB versus BA, BB and AA task [7]. This finding is even more remarkable given a recent suggestion that the idiosyncrasy in [7]’s (and our) study might encourage participants to focus on the last stimulus in the sequence and thus get trapped in using a simpler rule [63]. It is, thus, possible that non-human great apes—like humans—benefit from the presence of logical framing of sequences in SDL tasks. Improvements in the logical framing of the task could lead to even better performance (electronic supplementary material, S4). However, given our small sample size, this interpretation should be considered in the light of potential selection biases, i.e. Masindi’s remarkable learning and the performance differences between conditions may not only reflect the cognitive benefits of causal framing but also individual differences in disposition, motivation and prior experience (see STRANGE framework [64]). Masindi was the youngest chimpanzee in our sample, which might have benefited motivation and attentional focus. Two other chimpanzees in the causal cue condition (Eva and Kilimi) had been among the most reliable participants in this (and other concurrent) studies, suggesting that they may have been especially motivated and had accumulated more research experience. In contrast, one of the most motivated chimpanzees in the no-causal frame condition (Velu) was an adolescent who tended to get frustrated easily when he found the task to be challenging, which—together with his increasing interest in the social dynamics of the group—might have contributed to him gradually losing interest in the task. Such idiosyncratic factors, ranging from motivation, age, temperament and research experience, may have influenced task adherence and performance. Future studies with larger and more systematically characterized samples will be crucial to determine to what extent the observed effects generalize beyond a few particularly responsive individuals (see also [65]). As we did not test any monkeys in the causal frame condition test phase, we currently do not know whether causal sequences would have a similar boosting effect on SDL in monkeys. More data are needed to clarify to what extent non-human primates can process sequences quickly when the task is framed in a way that makes the relevance of sequencestransparent.

The data from the monkeys highlight some additional, non-sequencing challenges to our task. Only two out of seven squirrel monkeys passed training phase 1, in which only single events on a transparent tube were shown (but all capuchin monkeys passed). This might be due to a high demand for inhibiting a prepotent response (i.e. pointing to the location of the food reward): in trials in which the tube was empty and food was dropped into the tube, the food fell into the opaque bottom box of the apparatus but was still on the table (albeit invisible). Monkeys understand object permanence [53], which is beneficial for solving those phases in which the tube is opaque (training phase 2, test phase), but when it comes to understanding that food inside the bottom box is irretrievable, monkeys’ ability to remember and represent its location could have been detrimental to performance. Additionally, using food directly on the apparatus may have created a strong focus on the food, possibly interfering with performance.

Monkeys’ performance in training phase 2—in which the tube was made opaque before showing a single action and which only one capuchin monkey passed—highlights the demand on ‘general’ memory (i.e. not specific to sequential information): participants needed to remember the start state of the tube (empty or blocked) while watching the demonstration on the occluded tube and possibly also simulate what happened inside the tube after insertion of the item (towel or reward). While the memory for stimulus sequence hypothesis also highlights memory as a key cognitive bottleneck, it proposes species differences in a specific memory *for sequences* [2,61]. We acknowledge the possibility of interpreting training phase 2 as a sequencing task itself (in which case, however, one capuchin monkey passed it relatively quickly, within 108 trials (no data for chimpanzees for this phase; for further discussion see electronic supplementary material, S4)).

The SDL data with children showed that without causal information, children generally found the task difficult, suggesting that humans are not equipped with a strong proclivity to attend to sequences from early on in development. The causal frame condition showed that processing sequences *per se* was not difficult (at least not for children from 4 years of age): when children were provided with a causal frame in the training phase (phase 2, demonstrating single actions), they outperformed same-aged children in the no-causal condition (in both the training and test phase) and were more likely to correctly explain the rules of the task at the end of the study because causal connections between stimuli and the outcome helped children differentiate between sequence types. For children in the no-causal, versus causal, frame condition the ‘jump’ to inferring that the task involved a sequence of items was larger. It was only at 11 years of age that children were able to infer that the task was about sequences in the absence of a causal frame. This could have been the result of older children having accumulated more cultural experience with processing sequential information, being better able to use causal and temporal verbal labels in their reasoning (e.g. ‘*first* the paper towel has to go in’, ‘if there is a paper towel, *then* the token won’t fall’), improvements in counterfactual thinking (e.g. ‘if there was no paper towel, the token would fall, but if …’) and analogical reasoning, all of which change children’s hypotheses space when faced with our task. Moreover, with age children show (culturally and genetically driven) improvements in working memory capacity and may become better at inferring the experimenter’s intention. Another, compatible, explanation is that humans have a genetically based bias to attend to sequences and extract patterns from them (*dendrophilia hypothesis* [6]) which matures only later in childhood.

While we have found an impressive difference in performance between our chimpanzees and the bonobos undertaking an arbitrary SDL task [7], the comparison between the chimpanzee and human data still shows a large gap in SDL between humans and other animals [5,7], while suggesting the gap has been overestimated. We found tentative evidence that non-human great apes could hold a ‘middle position’ in SDL performance between humans and other animals, but clearly more data are needed to test this. It thus remains an open question to what extent the differences between humans and chimpanzees can be accounted for by differences specifically in how sequences are being processed [5] or in a more general memory capacity [15–17]. Proponents of the memory for stimulus sequence hypothesis themselves argue that animals have relatively poor memory for single arbitrary stimuli (see delayed MTS tasks [66]). Other, not mutually exclusive, theories are that humans have a unique inclination (if not capacity) to extract pattern information such as hierarchical structures from sequential information [6,62], a unique ability to encode and compress structures [4] or an enhanced general information processing capacity [3]. Future work may fruitfully test these theories with a broad set of tasks, looking at both sequence perception and production, across different species, and—an aspect that is missing so far—different cultural contexts.

We conclude that the idea that animals have a ‘limited capacity to discriminate ordered sequences of stimuli’ [2, p. 1] is premature, as the data are only based on arbitrarily structured sequences and limited regarding non-human great apes. This not only invalidates generalizations about processing of all kinds of sequences; such tasks seem to favour the engagement of human-specific experiences to decode and thus put humans and other animals on different starting points to the task, turning these ‘arbitrary’ sequencing tasks into tools to measure an individual’s *proclivity* to attend to sequential information rather than their *capacity* to do so (see *dendrophilia* versus *dendrocompetence* account [6]). While we have not refuted the memory for stimulus sequences hypothesis, i.e. the possibility that humans have genetically based enhanced sequence processing skills, we propose that for further evaluation more attention should be given to the nature of abstract cognitive tasks and to the design of tasks with greater ecological validity, particularly where comparisons between humans and non-human species are involved.

## 5. Methods

All studies and hypotheses were preregistered (links can be found in electronic supplementary material, S2.1).

### 5.1. Participants

We obtained valid data from 13 chimpanzees (seven *Pan troglodytes verus*, six hybrid), 24 brown tufted capuchin monkeys (*Sapajus apella*) and 24 squirrel monkeys (*Saimiri sciureus*) housed at the Royal Zoological Society of Scotland’s Edinburgh Zoo between July 2022 and December 2023, as well as from 77 English-speaking adult humans (tested online in March 2023) and 239 English-speaking children tested in the United Kingdom (aged 3−11 years, tested in person between July and September 2023). Participation was voluntary, and individuals could stop participating at any time. No participant was food- or water-deprived. The study received ethical approval from the University of St Andrews Teaching and Research Ethics Committee (PS15933), the Animal Welfare Ethical Review Board of Durham University, UK and the Living Links to Human Evolution Research Centre at the RZSS Edinburgh Zoo. For participant details see electronic supplementary material, S2.2.

### 5.2. Procedure

Non-human primates and children were tested in person. Materials were placed on a table between the experimenter and the participant, who was separated from the materials by a polycarbonate window with two small holes that they could use to indicate their choice by pointing or gesturing. Human adults were tested online, watching a video of the task with the set-up matched closely to that of the other groups. Animals completed multiple sessions per study phase, while humans completed the study in one session.

### 5.3. Choice (opt-out) training

All participants experienced a choice training phase (*opt-out training*) to familiarize them with the basic structure of the task (i.e. that they could adjust their choice according to the availability of a high value reward). They learned that in each trial they could choose between a green box (*opt-out box*) containing a low-value reward and a white box that sometimes contained a better reward (high-value food (animals), a large sticker (children) or 20-point tokens (human adults)). Prior to this training, food preference tests were undertaken with the animals to establish low- and high-value foods (see OSF pages).

In each trial, participants were presented with the green opt-out box and a white box (left/right location counterbalanced between participants; electronic supplementary material, figure S1). The experimenter opened the white box to reveal its contents (empty or high value reward) and then closed the box. Then the participant could point to one of the boxes. The opt-out box always contained a low-value reward; the white box contained a high-value reward in half of the trials. If there was a high-value reward inside the white box, the correct response was to choose the white box, and if empty, the correct response was to ‘opt out’ and choose the green box. Once the participant chose one of the boxes, the experimenter opened that box, revealed its contents and passed any reward to the participant. If the participant chose the white box when empty, the experimenter opened it and shook it a few times to demonstrate that it was empty. Humans received four to eight trials depending on performance; non-human animals a maximum of 10 sessions with 16 trials per session and had to reach a learning criterion of two consecutive sessions with 80% of trials per session (i.e. 13/16 trials) correct to proceed.

### 5.4. Training phases

Participants were semi-randomly assigned to the *causal frame* or *no-causal frame* condition (to ensure comparable sample sizes between conditions). Before introducing the SDL task, we conducted training phases to (i) familiarize participants with the characteristics of the RABS task and the number and type of physical objects involved to ensure that they were best prepared to use the causal frame provided. This was especially important as for practical reasons participants did not manipulate the objects before or during the study. The training was also conducted to (ii) check whether participants were able to correctly discriminate between *single* actions (i.e. A or B) so that in case of failure in the SDL task we could better assess whether participants specifically struggled with the sequencing part.

Monkeys received two training phases, chimpanzees only phase 1 and humans only phase 2 due to time restrictions. In both training phases, participants were presented with the opt-out box and the RABS apparatus, consisting of a transparent, rather than opaque, tube mounted on an opaque box, standing next to each other (figure 1). The left/right location of the apparatus and opt-out box were the same throughout the training and test phases within individuals but was counterbalanced between individuals within a condition. At the start of the trial, participants had a few seconds to look at the content of the tube, which was either empty or contained one towel as a blockage, depending on sequence type. In phase 1, the experimenter then demonstrated one action on the tube of the apparatus (insert towel or insert reward); in phase 2, the experimenter occluded the tube, making it opaque (as would be the case for the test phase), before showing the demonstration. Phase 2 familiarized participants with the requirement to hold information in mind (i.e. whether the tube was empty or contained a blockage), while the experimenter demonstrated an action, and to attend to the experimenter’s action to be able to infer the end state of the tube and make the correct choice.

The demonstration consisted of either inserting a reward into the tube or a paper towel (serving as blockage). The combination of the two start states (empty tube and towel in tube) and the two actions (insert reward and insert towel) resulted in four sequence types (table 2).

**Table 2.** Sequence types in the RABS training phases. RABS, reward and blockage sequence.

sequence type	content of tube at start of trial	occlusion of tube (only training phase 2)	action	correct choice
opt-out trial	tube empty	occlusion of tube	insert reward	green opt-out box
	tube empty	occlusion of tube	insert paper towel	
	paper towel in tube	occlusion of tube	insert paper towel	
high-value reward available trial	paper towel in tube	occlusion of tube	insert reward	RABS apparatus

After the demonstration, participants could choose between the opt-out box and the apparatus. For animals, a ‘no choice’ was treated as if having chosen the opt-out box (see electronic supplementary material). In the *causal frame* condition, if the participant pointed towards the apparatus, the experimenter lifted the tube from the box and turned it upside down. In high-value reward trials, the tube would contain a towel and a reward resting on top such that tipping the tube resulted in the reward falling out, which the experimenter gave to the participant. In opt-out trials, the tube was either empty or contained one or two towels. In these trials, if participants pointed to the apparatus, the experimenter would also lift and tip the tube, but this would not result in the delivery of a reward.

In the *no-causal frame* condition, when participants pointed to the apparatus, the experimenter would not detach the tube. Instead, they would open a small white box (a mini version of the white box used in the opt-out training) that was placed directly in front of the apparatus. This box would contain a reward only in high-value reward trials, i.e. where the tube contained a towel and a reward resting on top. The connection between the configuration of the tube and the delivery of the reward was thus less transparent as there was no physically logical reason why the specific configuration of ‘paper towel in tube and reward resting on top’ should be connected to the delivery of a reward.

For the animals, both training phases consisted of a maximum of 10 sessions, with each session consisting of 18 trials (nine high-value reward and nine opt-out). To proceed from training phase 1 to 2 and from training phase 2 to the test phase, animals needed to score 75% of the high-value reward trials and 75% of the opt-out trials per session correct in two consecutive sessions. For humans, who only received training phase 2, the training consisted of 12 trials for adults and six trials for children (adults: six high-value reward trials and two of each of the three opt-out sequence types; children: three high-value reward trials and one of each of the three opt-out trials). Humans proceeded to the test phase regardless of performance in the training (as the training was very short). For details see electronic supplementary material, S2.5.

### 5.5. Test

In the test phase, the tube was opaque from the beginning of each trial. The inside of the tube was never shown to participants before or during the demonstration. Therefore, participants were required to attend to the experimenter’s actions and their order to identify in which trials they could receive a reward by choosing the apparatus. In each trial, the experimenter demonstrated a sequence of two actions, after which participants could point to the apparatus or the opt-out box. There were four sequence types, one for each possible combination of actions (figure 1): AB, AA, BB or BA (A being ‘insert paper towel into tube’ and B being ‘insert reward into tube’). Pointing to the apparatus led to the delivery of a reward only after an AB sequence. To obtain as many rewards as possible, participants should choose the apparatus after seeing an AB sequence and the opt-out box after any of the other three sequences.

For the animals, the test phase consisted of a maximum of 25 sessions, with each session consisting of 12 trials (two blocks of six trials, with each block consisting of three AB trials and one each of the three other sequence types), totalling 300 test trials. Trial order per session was the same across participants. For human participants, to keep the study short and motivating, the test phase consisted of a single session of 12 trials, with trial order fixed across participants (electronic supplementary material, table S16). At the end of the test phase, adults were shown their total score (payment was not linked to the total score), asked questions regarding their attention level and any technical difficulties and were debriefed. Children were asked how they solved the game, collected the stickers they had earned, and parents/caregivers were debriefed.

### 5.6. Coding and analysis

For each trial, we coded whether the choice was correct and calculated the average proportion of correct trials per session split by sequence type (AB, AA, BB and BA) as well as across sequence types. To determine *successful discrimination*, we calculated a discrimination score (ranging from −1 to 1; electronic supplementary material, S2.7.1). We determined as ‘successful discrimination’ achieving a ratio at 0.67 or above in two consecutive sessions. This meant that within a test session a participant had to score all six opt-out trials correct as well as four of the six AB trials. The probability of doing so by chance performance is only 0.54% (see electronic supplementary material, table S17 and S2.7.1 for the coding rationale). Statistical analyses were carried out in R v. 4.3.2 [6,67]. For information on exploratory analyses and reliability coding see electronic supplementary material, S2.7 and S2.8.

## Data Availability

All data are available on the OSF page associated with this project [68]. Supplementary material is available online [69].

## References

[1] Fitch WT, Martins MD. 2014 Hierarchical processing in music, language, and action: Lashley revisited. Ann. NY Acad. Sci. **1316**, 87–104. (10.1111/nyas.12406) 24697242 PMC4285949

[2] Ghirlanda S, Lind J, Enquist M. 2017 Memory for stimulus sequences: a divide between humans and other animals? R. Soc. Open Sci. **4**, 161011. (10.1098/rsos.161011) 28680660 PMC5493902

[3] Cantlon JF, Piantadosi ST. 2024 Uniquely human intelligence arose from expanded information capacity. Nat. Rev. Psychol. **3**, 1–19. (10.1038/s44159-024-00283-3)

[4] Dehaene S, Al Roumi F, Lakretz Y, Planton S, Sablé-Meyer M. 2022 Symbols and mental programs: a hypothesis about human singularity. Trends Cogn. Sci. **26**, 751–766. (10.1016/j.tics.2022.06.010) 35933289

[5] Enquist M, Ghirlanda S, Lind J. 2023 The human evolutionary transition: from animal intelligence to culture. Princeton, NJ: Princeton University Press. (10.23943/princeton/9780691240770.001.0001)

[6] Fitch WT. 2018 What animals can teach us about human language: the phonological continuity hypothesis. Curr. Opin. Behav. Sci. **21**, 68–75. (10.1016/j.cobeha.2018.01.014)

[7] Lind J, Vinken V, Jonsson M, Ghirlanda S, Enquist M. 2023 A test of memory for stimulus sequences in great apes. PLoS One **18**, e0290546. (10.1371/journal.pone.0290546) 37672549 PMC10482264

[8] Wurz S. 2024 Becoming human: expert sequential and flexible thinking led to cumulative culture. South Afr. J. Sci. **120**, 17065. (10.17159/sajs.2024/17065)

[9] Heyes C. 2018 Cognitive gadgets. Cambridge, MA: Harvard University Press.

[10] Heyes C. 2020 Psychological mechanisms forged by cultural evolution. Curr. Dir. Psychol. Sci. **29**, 399–404. (10.1177/0963721420917736)

[11] Read DW, Manrique HM, Walker MJ. 2022 On the working memory of humans and great apes: strikingly similar or remarkably different? Neurosci. Biobehav. Rev. **134**, 104496. (10.1016/j.neubiorev.2021.12.019) 34919985

[12] Cheney DL, Seyfarth RM, Silk JB. 1995 The responses of female baboons (Papio cynocephalus ursinus) to anomalous social interactions: evidence for causal reasoning? J. Comp. Psychol. **109**, 134–141. (10.1037//0735-7036.109.2.134) 7758289

[13] Jensen G. 2017 Serial learning. In APA handbook of comparative psychology: perception, learning, and cognition (eds J Call, GM Burghardt, IM Pepperberg, CT Snowdon, T Zentall), pp. 385–409. Washington, DC: American Psychological Association. (10.1037/0000012-018)

[14] Howard-Spink E, Hayashi M, Matsuzawa T, Schofield D, Gruber T, Biro D. 2024 Nonadjacent dependencies and syntactic structure of chimpanzee action during a natural tool-use task. bioRxiv 2024-03. (10.1101/2024.03.25.586385) PMC11625446 39650560

[15] D’Amato MR, Salmon DP. 1984 Processing of complex auditory stimuli (tunes) by rats and monkeys (Cebus apella). Anim. Learn. Behav. **12**, 184–194. (10.3758/BF03213141) 6744117

[16] Bauer PJ, Shore CM. 1987 Making a memorable event: effects of familiarity and organization on young children’s recall of action sequences. Cogn. Dev. **2**, 327–338. (10.1016/S0885-2014(87)80011-4)

[17] Renner E, Price EE, Subiaul F. 2016 Sequential recall of meaningful and arbitrary sequences by orangutans and human children: does content matter? Anim. Cogn. **19**, 39–52. (10.1007/s10071-015-0911-z) 26298671

[18] Bauer PJ, Fivush R. 1992 Constructing event representations: building on a foundation of variation and enabling relations. Cogn. Dev. **7**, 381–401. (10.1016/0885-2014(92)90023-k)

[19] Dedhe AM, Piantadosi ST, Cantlon JF. 2023 Cognitive mechanisms underlying recursive pattern processing in human adults. Cogn. Sci. **47**, e13273. (10.1111/cogs.13273) 37051878 PMC11097651

[20] Albiach-Serrano A, Sebastián-Enesco C, Seed A, Colmenares F, Call J. 2015 Comparing humans and nonhuman great apes in the broken cloth problem: is their knowledge causal or perceptual? J. Exp. Child Psychol. **139**, 174–189. (10.1016/j.jecp.2015.06.004) 26117496

[21] Albiach-Serrano A, Call J. 2014 A reversed-reward contingency task reveals causal knowledge in chimpanzees (Pan troglodytes). Anim. Cogn. **17**, 1167–1176. (10.1007/s10071-014-0749-9) 24744182

[22] Hanus D, Call J. 2011 Chimpanzee problem-solving: contrasting the use of causal and arbitrary cues. Anim. Cogn. **14**, 871–878. (10.1007/s10071-011-0421-6) 21647648

[23] Haun DBM, Call J. 2009 Great apes’ capacities to recognize relational similarity. Cognition **110**, 147–159. (10.1016/j.cognition.2008.10.012) 19111286

[24] Jordan EJ, Townrow LAJ, Wright CI, Seed AM. 2020 Understanding solidity: investigating knowledge of a functional object property in brown capuchin monkeys (Sapajus apella) and common squirrel monkeys (Saimiri sciureus). Anim. Behav. Cogn. **7**, 365–391. (10.26451/abc.07.03.07.2020)

[25] Mayer C, Call J, Albiach-Serrano A, Visalberghi E, Sabbatini G, Seed A. 2014 Abstract knowledge in the broken-string problem: evidence from nonhuman primates and pre-schoolers. PLoS One **9**, e108597. (10.1371/journal.pone.0108597) 25272161 PMC4182709

[26] Seed AM, Call J. 2014 Space or physics? Children use physical reasoning to solve the trap problem from 2.5 years of age. Dev. Psychol. **50**, 1951–1962. (10.1037/a0036695) 24773103

[27] Premack D. 1983 The codes of man and beasts. Behav. Brain Sci. **6**, 125–136. (10.1017/s0140525x00015077)

[28] Kroupin IG, Carey SE. 2022 You cannot find what you are not looking for: population differences in relational reasoning are sometimes differences in inductive biases alone. Cognition **222**, 105007. (10.1016/j.cognition.2021.105007) 34990990

[29] Kroupin IG, Carey SE. 2022 The importance of inference in relational reasoning: relational matching as a case study. J. Exp. Psychol. **151**, 224–245. (10.1037/xge0001068) 35238601

[30] Kroupin I. 2021 Inference in relational reasoning: a case study of relational-match-to-sample. Doctoral dissertation, Harvard University, Cambridge, MA.

[31] Christie S. 2021 Learning sameness: object and relational similarity across species. Curr. Opin. Behav. Sci. **37**, 41–46. (10.1016/j.cobeha.2020.06.010)

[32] Felsche E, Völter CJ, Herrmann E, Seed AM, Buchsbaum D. 2024 How can I find what I want? Can children, chimpanzees and capuchin monkeys form abstract representations to guide their behavior in a sampling task? Cognition **245**, 105721. (10.1016/j.cognition.2024.105721) 38262272

[33] Hochmann JR, Tuerk AS, Sanborn S, Zhu R, Long R, Dempster M, Carey S. 2017 Children’s representation of abstract relations in relational/array match-to-sample tasks. Cogn. Psychol. **99**, 17–43. (10.1016/j.cogpsych.2017.11.001) 29132016

[34] Goddu MK, Lombrozo T, Gopnik A. 2020 Transformations and transfer: preschool children understand abstract relations and reason analogically in a causal task. Child Dev. **91**, 1898–1915. (10.1111/cdev.13412) 32880903

[35] Katz JS, Wright AA, Bachevalier J. 2002 Mechanisms of same-different abstract-concept learning by rhesus monkeys (Macaca mulatta). J. Exp. Psychol. **28**, 358–368. (10.1037//0097-7403.28.4.358) 12395493

[36] Wasserman E, Castro L, Fagot J. 2017 Relational thinking in animals and humans: from percepts to concepts. In APA handbook of comparative psychology: vol 2. Perception, learning, and cognition (eds J Call, GM Burghardt, IM Pepperberg, CT Snowdon, T Zentall), pp. 359–384. Washington, DC: American Psychological Association. (10.1037/0000012-000)

[37] Wright AA, Kelly DM, Katz JS. 2021 Same/different concept learning by primates and birds. Learn. Behav. **49**, 76–84. (10.3758/s13420-020-00456-z) 33742425

[38] Wright AA, Rivera JJ, Katz JS, Bachevalier J. 2003 Abstract-concept learning and list-memory processing by capuchin and rhesus monkeys. J. Exp. Psychol. **29**, 184–198. (10.1037/0097-7403.29.3.184) 12884678

[39] Christie S, Gentner D, Call J, Haun DBM. 2016 Sensitivity to relational similarity and object similarity in apes and children. Curr. Biol. **26**, 531–535. (10.1016/j.cub.2015.12.054) 26853364

[40] Golubeva IY, Tikhonravov DL, Kuznetsova TG. 2024 Different cognitive strategies for determining common image features in other primates and preschool children. Int. J. Primatol. **45**, 336–359. (10.1007/s10764-023-00403-5)

[41] Martin-Ordas G. 2022 Spontaneous relational and object similarity in wild bumblebees. Biol. Lett. **18**, 20220253. (10.1098/rsbl.2022.0253) 36043304 PMC9428533

[42] Rakoczy H, Haun D. 2020 Comparative cognition between children and animals. In The encyclopedia of child and adolescent development (eds S Hupp, J Jewell). Hoboken, NJ: John Wiley & Sons. (10.1002/9781119171492)

[43] Rosati AG, Wobber V, Hughes K, Santos LR. 2014 Comparative developmental psychology: how is human cognitive development unique? Evol. Psychol. **12**, 448–473. (10.1177/147470491401200211) 25299889 PMC10481050

[44] Barton RA, Venditti C. 2014 Rapid evolution of the cerebellum in humans and other great apes. Curr. Biol. **24**, 2440–2444. (10.1016/j.cub.2014.08.056) 25283776

[45] MacLeod CE, Zilles K, Schleicher A, Rilling JK, Gibson KR. 2003 Expansion of the neocerebellum in Hominoidea. J. Hum. Evol. **44**, 401–429. (10.1016/s0047-2484(03)00028-9) 12727461

[46] Weisman RG, Wasserman EA, Dodd PW, Larew MB. 1980 Representation and retention of two-event sequences in pigeons. J. Exp. Psychol. **6**, 312–325. (10.1037//0097-7403.6.4.312)

[47] Farrar MJ, Goodman GS. 1992 Developmental changes in event memory. Child Dev. **63**, 173–187. (10.1111/j.1467-8624.1992.tb03605.x) 1551325

[48] Hudson J, Nelson K. 1983 Effects of script structure on children’s story recall. Dev. Psychol. **19**, 625–635. (10.1037//0012-1649.19.4.625)

[49] Bauer PJ, Mandler JM. 1989 One thing follows another: effects of temporal structure on 1- to 2-year-olds’ recall of events. Dev. Psychol. **25**, 197–206. (10.1037//0012-1649.25.2.197)

[50] Bauer PJ, Mandler JM. 1992 Putting the horse before the cart: the use of temporal order in recall of events by one-year-old children. Dev. Psychol. **28**, 441–452. (10.1037//0012-1649.28.3.441)

[51] Whiten A, Flynn E, Brown K, Lee T. 2006 Imitation of hierarchical action structure by young children. Dev. Sci. **9**, 574–582. (10.1111/j.1467-7687.2006.00535.x) 17059454

[52] Wilson B, Spierings M, Ravignani A, Mueller JL, Mintz TH, Wijnen F, van der Kant A, Smith K, Rey A. 2020 Non‐adjacent dependency learning in humans and other animals. Top. Cogn. Sci. **12**, 843–858. (10.1111/tops.12381) 32729673 PMC7496455

[53] Cacchione T, Rakoczy H. 2017 Comparative metaphysics: thinking about objects in space and time. In APA handbook of comparative psychology: perception, learning, and cognition (eds J Call, GM Burghardt, IM Pepperberg, CT Snowdon, T Zentall), pp. 579–599, vol. 2. Washington, DC: American Psychological Association. (10.1037/0000012-026)

[54] Call J. 2004 Inferences about the location of food in the great apes (Pan paniscus, Pan troglodytes, Gorilla gorilla, and Pongo pygmaeus). J. Comp. Psychol. **118**, 232–241. (10.1037/0735-7036.118.2.232) 15250810

[55] Call J. 2007 Apes know that hidden objects can affect the orientation of other objects. Cognition **105**, 1–25. (10.1016/j.cognition.2006.08.004) 17026977

[56] Santos LR, Hauser MD. 2002 A non‐human primate’s understanding of solidity: dissociations between seeing and acting. Dev. Sci. **5** 1–7. (10.1111/1467-7687.t01-1-00216)

[57] Schloegl C, Waldmann MR, Fischer J. 2013 Understanding of and reasoning about object–object relationships in long-tailed macaques? Anim. Cogn. **16**, 493–507. (10.1007/s10071-012-0591-x) 23417558 PMC3625412

[58] Amici F, Aureli F, Call J. 2010 Monkeys and apes: are their cognitive skills really so different? Am. J. Phys. Anthropol. **143**, 188–197. (10.1002/ajpa.21305) 20853474

[59] de Blois ST, Novak MA, Bond M. 1998 Object permanence in orangutans (Pongo pygmaeus) and squirrel monkeys (Saimiri sciureus). J. Comp. Psychol. **112**, 137–152. (10.1037//0735-7036.112.2.137) 9642783

[60] Baayen RH. 2008 Analyzing linguistic data. a practical introduction to statistics using R. Cambridge, UK: Cambridge University Press.

[61] Lind J, Jon-And A. 2024 A sequence bottleneck for animal intelligence and language? Trends Cogn. Sci. **29**, 2024. (10.1016/j.tics.2024.10.009) 39516147

[62] Tecumseh Fitch W. 2018 Bio-linguistics: monkeys break through the syntax barrier. Curr. Biol. **28**, R695–R697. (10.1016/j.cub.2018.04.087) 29920260

[63] Zentall TR, Peng DN. 2024 The problem with two-event sequence learning by pigeons. Anim. Cogn. **27**, 63. (10.1007/s10071-024-01906-1) 39361035 PMC11450055

[64] Webster MM, Rutz C. 2020 How STRANGE are your study animals? Nature **582**, 337–340. (10.1038/d41586-020-01751-5) 32541916

[65] Bandini E, Girish S, Barnes P, Boot M, Tennie C, Forss S. 2025 The Biases in Captive Chimpanzee Cognitive Research: First Insights From the Ape Research Index (ARI Database. Ethology (10.1111/eth.70007)

[66] Lind J, Enquist M, Ghirlanda S. 2015 Animal memory: a review of delayed matching-to-sample data. Behav. Process. **117**, 52–58. (10.1016/j.beproc.2014.11.019) 25498598

[67] R Core Team. 2024 R: a language and environment for statistical computing. Vienna, Austria: R Foundation for Statistical Computing. See https://www.R-project.org/.

[68] Reindl E, Kendal R, Seed A, Barton R. 2025 The role of causal cues for sequence discrimination learning in primates. OSF https://osf.io/4nt89/

[69] Reindl E, Seed A, Barton R, Francis-Costa T, Kendal R. 2025. Supplementary Material from: Humans May Not Have a Uniquely Enhanced Sequence Memory: Sequence Discrimination Is Facilitated by Causal-Logical Framing in Humans and Chimpanzees. FigShare. (10.6084/m9.figshare.c.7881973) PMC12303101 40727398

